# Loneliness and Daily Memory Lapses in Older Adults: Findings From the Einstein Aging Study

**DOI:** 10.1093/geroni/igaf122.461

**Published:** 2025-12-31

**Authors:** Jee eun Kang, Christopher Engeland, Martin Sliwinski, Jennifer Graham-Engeland

**Affiliations:** The Pennsylvania State University, University Park, Pennsylvania, United States; The Pennsylvania State University, University Park, Pennsylvania, United States; The Pennsylvania State University, University Park, Pennsylvania, United States; The Pennsylvania State University, University Park, Pennsylvania, United States

## Abstract

Although loneliness is a known risk factor for cognitive decline, the degree to which it predicts subjective cognitive functioning—such as daily memory lapses—is unclear. Moreover, memory difficulties themselves may contribute to feelings of loneliness by disrupting social interactions and diminishing confidence in cognitive abilities. This study utilized ecological momentary assessment (EMA) data to examine the bidirectional daily associations between loneliness and self-reported prospective (PM) and retrospective memory (RM) lapses. A sample of 263 older adults (mean age = 77 years, 67% women, 43% Black, 31% MCI) completed 14 days of EMA, reporting momentary loneliness (5×/day) and daily memory lapses each evening. Multilevel models tested the effects of loneliness on same-day and next-day memory lapses and vice versa, controlling for study day, age, gender, education, MCI status, marital status, living arrangement, and prior-day outcomes. On days when participants felt lonelier than usual, they were more likely to forget meetings (OR = 1.07, p=.052), although this effect was marginally significant; moreover, forgetting meetings predicted higher loneliness the next day (b = 4.2, p=.003). Further, greater irritation from PM lapses was associated with higher loneliness on the same (b=.03, p=.020) and next day (b=.03, p=.04). Higher loneliness was also associated with forgetting important information (OR = 1.03, p=.049) on the same day and misplacing items the next day (OR = 1.03, p=.010). Findings highlight a potentially bidirectional relationship, suggesting that loneliness contributes to daily cognitive difficulties, which, in turn, may reinforce loneliness. Addressing memory lapses as well as loneliness may help support cognitive and emotional well-being in aging populations.

